# Quarterly Summary of the Transactions of the College of Physicians of Philadelphia, for May, June, and July

**Published:** 1842-08-06

**Authors:** 


### MEDICAL EXAMINER.

NEW SERIES.

No. 32.] PHILADELPHIA, AUGUST 6, 1842. [Vol. I.

### BIBLIOGRAPHICAL NOTICES.

*Quarterly Summary of the Transactions of the College of Physicians of Philadelphia, for May, June and July.*

The third number of this little journal is before us, and, in point of practical value, takes precedence of both its predecessors. It is to be wished, however, that the proof sheets should undergo more rigid scrutiny. In our notice of the first number, the errors of the press were made a subject of censure, and in the present pamphlet great neglect is observable. It is asking too much, both of the author and the reader, to compel them to decipher passages in which an entire line is transposed from the foot of one page to that of the next preceding. (See pages 68 and 69.)

At the session of May 3d, Dr. Isaac Parrish read a paper upon two interesting cases of fatal invagination of the lower portion of the colon into the rectum, occurring in twin children aged about six months. The symptoms and duration of suffering in both cases were identical:—violent abdominal pain, feculent and bloody discharges, and death in about thirty-six hours. About the same interval elapsed between the death of the first and the attack of the second child.

The report of the verbal communication of Dr. D. Francis Condie forms, perhaps, the most important part of the contents of the number. It gives an account of the peculiar and probably contagious epidemic puerperal fever, prevalent in and about Philadelphia for some months preceding the date of the meeting, though now happily past. The question of contagion, and that of the congeneric character of puerperal fever and malignant erysipelas, constitute the most interesting points in the communication and the subsequent discussion. The latter question has been much agitated in Europe, and may even be regarded as so far settled by former experience in this city, that we ventured, many years ago, before the Philadelphia Medical Society, and in surgical lectures, to impress the importance of modifying the treatment of traumatic injuries whenever puerperal fever makes its appearance epidemically, and using every precaution in anticipation of disastrous confinements whenever erysipelas becomes prevalent. As the circulation of the journal of the College is not extended with all the energy devoted to the distribution of periodicals which are expected to yield a pecuniary profit, we shall do our readers no injustice in making liberal extracts from the report under notice.

"Dr. Condie begged leave to call the attention of the College to a subject of very great interest to the medical profession, especially to those of its members engaged in obstetrical practice. He alluded, he remarked, to the prevalence, at the present time, of puerperal fever of a peculiarly insidious and malignant character. So far as his own observations and those of his medical associates with whom he had conversed on the subject, extended, nearly every case of the disease that had occurred up to this date, had terminated fatally. Dr. C. was unable to state positively the exact extent of the fever, either in regard to the number of parturient females who have been attacked by it, or the limits within which it had as yet been confined. The remarks he was about to offer, were founded upon cases that had occurred in the southern sections of the city, and neighbouring districts. In the practice of one gentleman, extensively engaged as an obstetrician, nearly every female he has attended in confinement, during several weeks past, within the above limits, had been attacked by the fever. It is also known that the disease has occurred in the lying-in wards of the Philadelphia Hospital, at Blockley, and the Doctor has heard of several cases that have occurred in the northern and western portions of the county.

"So far as the observations of Dr. C. extend, the disease has been found to occur alike in the young and middle aged—the robust and delicate—in those surrounded by every comfort and afforded every attention demanded by their situation, as in the poor and destitute—as well in those who were confined for the first time, as in those who had already borne a number of children—and as well after the most rapid and easy labours, as after those that were protracted and difficult.

"Usually, within the first three days, but sometimes within a few hours after delivery, the patient was seized with a chill, differing in intensity in different cases—being sometimes so slight as scarcely to attract attention, while at other times it amounted to a perfect rigour. The chill was quickly succeeded by a febrile reaction, attended with a hot, dry skin, some thirst, a white milky fur upon the tongue, and a quick, rapid pulse, amounting in some cases to 160 or 170 and upwards in a minute. The pulse was often full, but invariably soft and compressible. There was, from the very onset of the disease, a peculiar anxious or distressed expression of the countenance —and a mottled or irregular flushed appearance of the face. The patient soon after the attack, generally complained of some soreness or dull pain—often confined, at first, to the groins or across the hypo-gastric region. The pain was increased upon pressure. It very speedily increased in intensity, and spread over the whole of the abdomen, which now became tumid and more or less tympanitic.

"In many cases, the pain of the abdomen was described by the patient rather as a sense of soreness than of acute pain, and although augmented upon the slightest pressure being applied, the increased suffering was indicated more by the expression of the patient's countenance than by acute cries. Occasionally, the Doctor found that when the pressure was steadily continued, and gradually increased, the pain would appear to be rather lessened than augmented. The bowels were usually constipated, but in all the cases which fell under the observation of Dr. C., they were easily acted upon—the exhibition of an ordinary dose of any mild cathartic causing full and repeated evacuations, differing in appearance, being either ordinary fœces, or a dark coloured, very offensive fluid—followed, more or less speedily, by the frequent discharge, with considerable tenesmus, of small portions of a slightly discoloured mucus. In one case, in which the discharges from the bowels were very frequent during the entire continuance of the disease, they consisted entirely of small quantities of a transparent gelatiniform fluid.

"From the first onset of the disease the stomach was, in nearly every case, extremely irritable—rejecting soon after they were swallowed, the drinks and other articles taken by the patient. Occasionally, the frequent ejection, by vomiting, of a greenish flocculent fluid, occurred at an early period of the disease.

"Very generally, so soon as the disease became fully developed, the secretion of milk, as well as the lochial discharge, ceased, or became greatly diminished—occasionally, however, the milk was secreted very freely until within a few hours before death, and the only change observed in the condition of the lochia, was in its becoming much darker, and exhaling a disagreeable, but not decidedly fœtid odor.

"Soon after the occurrence of the abdominal pain and tumefaction, the respiration of the patient became short and oppressed, and attended with an intolerable sense of weight and uneasiness at the præcordia. Some of the patients referred all of their sufferings, in the advanced stage of the disease, to this peculiar and distressing sensation; declaring that if only this could, by any means, be removed, they would be well. The oppression and difficulty of respiration was not always found to be in direct proportion to the emphysematous distension of the abdomen.

"As the disease progressed, the abdomen became, in general, more swollen, tense, and painful; the shortness of respiration more striking, and the pulse more frequent, quick and feeble—the countenance of the patient assuming a very peculiar, dusky hue, and dejected expression. The irritability of the stomach increased—vomiting became frequent—and, very commonly, there speedily ensued a discharge from the stomach, at short intervals, by a species of eructation, of mouthfuls of a dark greenish or chocolate coloured, flocculent fluid, which, according to Dr. C.'s observation, was invariably a fatal symptom; it being very soon succeeded by a cold, clammy condition of the skin, occurring first in the extremities—a dark leaden hue and haggard expression of the countenance—a sunken state of the eyes—profuse perspiration, especially about the head, face, and superior extremities—and death. In one or two cases, death was preceded by violent delirium—an injected state of the eyes—flushing of the cheeks—convulsive movements of the limbs, and other symptoms of encephalic inflammation; but, in the majority of instances, the mental powers of the patient were but little affected throughout the disease; death appearing to result from complete exhaustion of the vital powers, and occurred without a struggle.

"In some of the cases, a short time previous to death, there occurred so complete a remission of all the distinguishing symptoms of the disease, as to induce the belief, even in the medical attendants, that the patient was about to recover. This remission was, however, in all the instances which have fallen under the notice of Dr. C., speedily followed by collapse and death. In one striking instance, seen by the Doctor, in consultation with Drs. Hewson and Hodge, the patient, on the fourth day after what was considered a very severe attack, was found sitting up in bed, free from fever, pain, and difficulty of breathing, nursing her child; on being questioned, she declared that she felt perfectly well, though weak—the next evening she was a corpse.

"Death generally occurred upon the third or fourth day of the disease—in but few cases was the disease protracted beyond the fifth day.

"The Doctor remarked, that it was a fact worthy of notice—though he was not aware it would throw much light upon the causes and pathological character of the disease—that in the neighbourhoods and even houses in which cases of puerperal fever have occurred, erysipelas has prevailed to a greater or less extent. Erysipelas has, indeed, been far more prevalent throughout the whole of the districts south of the city, during the past winter and spring, than Dr. C. has known it to be during the last twenty-six years."

Dr. Condie appears to have been convinced, from the facts under his notice, that this peculiar puerperal fever was decidedly contagious, and capable of being transmitted by the person or clothes of the physician.

"How otherwise can be explained, the very curious circumstance of the disease, in one district, being exclusively confined to the practice of a single physician, [Dr. Rutter,] a Fellow of this College, extensively engaged in obstetrical practice—while no instance of the disease has occurred in the patients under the care of any other accoucheur practising within the same district? Scarcely a female that has been delivered by this gentleman for weeks past has escaped an attack."

"The cases of the disease that have fallen under the notice of Dr. C., have been very variously treated; in the majority of them, the effects of venesection in the first stage, followed by active purgation, have been very fully tried, followed by fomentation and blisters to the abdomen, and Dover's powder, the nitrous powders with calomel, pills of blue mass, opium and ipecacuanha, spirits of turpentine, &c., internally. Under every variety of treatment the disease has appeared to run pretty much the same fatal course. So far as the observations of Dr. C. extend, the disease is not one in which active depletion, but more especially by the lancet, will be found to produce any good effects;—in fact, in no one of the cases in which he has been consulted, could he be induced even in the earliest stages, to give his consent to the detraction of blood to any extent—so strongly did the character of the pulse, and all the symptoms present contra-indicate it. In one instance the extensive application of leeches over the abdomen, appeared to him, to cause a very decided abatement of the pain, and more urgent symptoms of the disease; but from the rapidity with which the patient subsequently sunk, and the early period at which death took place, he is fearful that the occurrence of the fatal collapse, was accelerated by the leeching.

"In the cases of the disease which have occurred in the Philadelphia Hospital, Blockley, the effects of direct depletion have been very fully tried—in every instance, however, in which venesection has been resorted to, in that institution, if Dr. C. is correctly informed, the disease has terminated fatally.

"Blisters to the abdomen were certainly beneficial in every instance, by the abatement they procured of the pain and intumescence—applied over the præcordia, they appeared, in some instances, to relieve very decidedly the oppression and distress caused by the difficulty of respiration.

"In one case, the administration of small doses of acetate of lead in solution, suggested by Dr. Hewson, very speedily abated the irritability of the stomach, so as to enable it to retain the drinks and remedies subsequently administered, which previously had been almost immediately rejected."

In answer to a question from Professor Huston, Dr. C. remarked that the majority of the children lived and did well. Two children died of erysipelas; one shortly—the other three weeks after birth. He had declined witnessing autopsies from prudent regard to his own obstetric patients.

Dr. Rutter, some of whose cases were examined after death, referred the College to Dr. Ashmead, the operator, for particulars; and the following are the most important details.

"Dr. Ashmead had found in all of these three cases nearly the same lesions, differing only in degree. In the first case examined by him, there was general peritoneal inflammation, with slight effusion of serum with flocculi floating in it; serous infiltration in the cellular tissue of the broad ligaments, a little lymph on the surface of one of the ovaries, a rose colored blush covering the peritoneum of the uterus and intestines, no adhesion among the intestines, and great tympanitis. The uterus being laid open presented a perfectly natural appearance. In the second case, the patient had died on the sixth day. There was the same appearance of peritoneal inflammation, but in a higher degree, with serous effusion, and slight recent adhesions between the peritoneal surfaces of the intestines. Pus was found in the cellular tissue of the broad ligaments, in the structure of the uterus, and Dr. A. believed, also in the cavity of the veins—the uterine cavity was healthy. This patient had vomited a dark or coffee colored substance, a quantity of which was found in the stomach after death. In the third case, the patient had died on the third day. A large quantity of lymph was found effused in the cavity of the peritoneum, with a copious deposite of pus in the broad ligaments. Dr. Ashmead thought, that the veins were also involved in this case, but Dr. Hodge, who was present at the autopsy, did not consider the appearance sufficiently positive to substantiate this conclusion. In this, as well as in the other cases, the liver, spleen, and kidneys were softened, as is seen in cases of low, malignant fevers. In one of the cases, the stomach contained a fluid resembling coffee grounds, and probably the same as the black vomit of yellow fever; the follicles of the mucous membrane of the stomach were in this case enlarged, although its mucous surface was not inflamed."

The following remarks on the cases occurring in the Philadelphia Almshouse—where Dr. Condie seemed to regard the disease as of somewhat less malignant form than in the district where he resides—display, at least, some shades of difference between these cases and those of Dr. Rutter, and contain some curious illustrations of the affinities of the affection.

"Dr. Huston, rose to make some statements in reference to the appearauce of this alarming disease in the Philadelphia Hospital, with which institution he was connected as obstetrician. About two weeks since, he and his colleague, Dr. Gillingham, were sent for on account of the occurrence of the epidemic.

"Three or four cases had died under the treatment of the house pupils, and three cases were then pending.

"These had been largely depleted, and had taken active cathartics and enemata, without producing any action on the bowels, which appeared in all of them to be remarkably torpid. These cases also died.

"One woman had been delivered about twenty-four hours previous to their visit, and had just been seized with the disease ; her respiration was very bad, pulse rapid and compressible, tongue of a cream color, (which Dr. H. had noticed in all the cases,) restlessness extreme, face suffused, being red and blue in spots. These symptoms had been preceded by a heavy chill, and the case was considered a very severe one, in its incipient stage. Dr. Huston ventured to suggest a change in the practice, especially as all the other cases had died. He proposed a full dose of opium, (2 grs.) with calomel, sinapisms to the feet, carbonate of ammonia julep, &c.—no blood was abstracted from the arm—and under this plan the patient recovered.

"Dr. Huston was informed by Dr. Barron, the house pupil, that all the children, born of the women who had died of this epidemic, had died of peritoneal inflammation. The lying-in ward has since been cleared, and the patients removed to other parts of the house. Three women confined during the prevalence of the epidemic, escaped the disease. One of these was a cripple, who occupied a bed in the same room with those who had died. Dr. Huston has not seen the disease in private practice.

"Dr. H. is opposed to the use of the lancet in the epidemic. The condition of the nervous system is such as utterly to forbid it. It is from the first broken down by the poison producing the disease, and all the symptoms indicate an opposite course of treatment. The experience of this city, Dr. H. believes, does not justify the practice. In the present epidemic it has certainly failed, and in the only case of recovery which he has seen, it was not used. This corresponds with his observations in former years. Dr. H. has tried the antimonial practice, and believes it may answer a good purpose in mild sporadic cases, but not in these malignant cases. The epidemic and sporadic cases are altogether different.

"Dr. Huston has noticed a tendency to erysipelas, lately, and has seen three cases of severe illness in men, bearing an analogy to the disease under consideration. There was the same appearance of the tongue and pulse, the same violent abdominal pain, as in this epidemic. One of these patients had died—neither of them would bear the abstraction of blood."

Dr. Ashmead argued against the contagiousness of the disease, stating instances of its prevalence in certain country districts, where it did not follow in the track of any particular physician.

" Dr. Rutter observed, that after the occurrence of a number of cases of the disease in his practice, he had left the city, and remained absent for a week, but on returning, no article of clothing he then wore having been used by him before, one of the very first cases of parturition he had attended was followed by an attack of the fever, and terminated fatally—he cannot readily, therefore, believe in the transmission of the disease from female to female, by a contagion conveyed in the person or clothes of the physician."

Dr. Warrington had avoided being present at autopsies for fear of conveying the disease. He had not seen it in the malignant form described by Dr. Condie, but, in milder cases, his experience was in favour of active depletion. The following facts bearing on contagion, and the affinity with erysipelas—condensed from his remarks—are curious. He attended his first case in this epidemic in January last. After instrumental labour with alarming hæmorrhage, the patient had a rigor on the fifth day, and died of peritonitis, with flocculent, lymphy, and puruloid serous effusion, in five days from that time. A few days thereafter, he attended three cases in rapid succession. One of these had subsequent metritis; another, peritonitis confined to the surface of the womb;—both of these recovering, under free depletion,—and the third fell a victim to puerperal peritonitis, after free depletion, in five days from the attack. Two other cases, presented at short intervals, died in like manner, on the fifth day of illness, under similar treatment. The nurse of the last case was attacked with violent erysipelas of the head and face, in a few days after her patient's death, and also died; "she was not bled." In the early part of the succeeding month, two cases of disease, in great degree analogous, occurred in the practice of Dr. W., both of whom recovered under actively depletory treatment.

In reply to Dr. Ashmead's argument in favour of the non-contagious character of the disease, Dr. Condie dwelt upon the diminished liability to the transfer of contagion by the clothes or person of the country practitioner, whose cases rarely occur in rapid succession,—days, and weeks frequently intervening,—and whose rides are long and frequent through a pure and free atmosphere.

"He observed, moreover, that one fact would seem, however, to establish the eminently contagious or infectious character of the more virulent cases of puerperal fever, and which is, that where it occurs as an epidemic in the lying-in wards of an hospital, no other means have been found to prevent the spread of the disease among the inmates and check its further occurrence, except that of dispersing the patients and abandoning the wards. It has even happened that after the wards have been fully cleansed and ventilated, and remained vacant for some time, on their re-occupation the disease has broken out anew."

It may be well here to remark, that the old lying-in ward of the Pennsylvania Hospital, located in the east wing of the building, became so much infested with puerperal fever, for a series of years, that the physicians were obliged to transfer the patients into rooms in the centre building, and, in consequence, the disease immediately disappeared. On every attempt to re-occupy the original apartment with obstetrical cases, the mortality immediately recommenced, as though the cause of the affection adhered to the room with the pertinacity with which the poison of the glanders adheres to a stable in which it has once occurred. After a long time, the same obstinate tendency developed itself in the new apartments, and the patients were only protected by removal to another building. Yet in other diseases, the infected portions of the house did not prove in any degree objectionable for the accommodation of persons labouring under other complaints. On this subject, and on the cotemporaneous existence of erysipelas and puerperal fever, some very interesting illustrations were also offered by Dr. Stewardson and Dr. Henry H. Smith, drawn from observations made in the Pennsylvania Hospital; but perhaps the most curious evidence was that related by Dr. Samuel Jackson, formerly of Northumberland, to Dr. West, and, by him, to the College. It is as follows:

"Women," the doctor remarked, "who had expected me to attend upon them, now becoming alarmed, removed out of my reach, and others sent for a physician residing several miles distant. These women, as well as those attended by midwives, all did well; nor did we hear of any deaths in child-bed within a radius of fifty miles, excepting two, and those I afterwards ascertained to have been caused by other diseases. I now began to be seriously alarmed on the score of contagion. Although I had used some personal precautions before, I now feared that they had not been sufficient.

"To the next case of parturition, then, I did not go without, as was supposed, a thorough purification. This woman was attacked with the disease and died. I then recollected that I had worn to her house the same gloves, lined with flannel, which I had worn through all my previous attendance. I do not pretend to say that this was the source of the contagion in this case. I merely state the fact.

"During the next two months, I attended several obstetrical patients, who all did well; and then I was again horrified with a decisive and violent case of the fever. The patient recovered under the most vigorous depletion by bleeding, puking, sweating, and purging. A ninth case soon after occurred, and was cured by the same means.

"No contagion, it is certain, could have reached these last two cases, unless through the medium of injection pipes and bladders, which had been used by them a day or two after delivery, and which I found were the very instruments that had been used in two of my former cases, and then put away, probably without any purification.

"It may be worthy of remark, that the house in which the first case of the disease occurred, was filthy almost beyond comparison; add to which, I was attending and dressing a limb extensively mortified from erysipelas, and went immediately to the accouchement with my clothes and the unfortunate gloves most thoroughly imbued with the effluvia of that sphacelation. The erysipelas had been very prevalent for six months and difficult to manage.

"Never before nor since that time, have I seen any thing similar to this disease—nothing similar even to the four cases that recovered. The disease is, indeed, exceedingly characteristic, and may be recognised even by the countenance."

At the session of June 7th, Professor Samuel Jackson read the Annual Report on the theory and practice of Medicine. It dwells chiefly on the versatility of medical theories in a spirited and imaginative style; but it is to be regretted that the annual reports of the committees of the College are rarely papers of deep research, being too frequently regarded as mere matters of form.

